# Incidence of maxillary sinus septa in the saudi population

**DOI:** 10.1186/s12880-023-00980-0

**Published:** 2023-02-04

**Authors:** Amani Mirdad, Razan Alaqeely, Sumaiah Ajlan, Mazen A. Aldosimani, Nahid Ashri

**Affiliations:** 1 Department of Periodontics and Community Dentistry, College of Dentistry, King Saud University, Riyadh, Saudi Arabia; 2 Department of Oral Medicine and Diagnostic Sciences, College of Dentistry, King Saud University, Riyadh, Saudi Arabia

**Keywords:** Sinus septa, Maxillary sinus, CBCT, Incidence, Saudi

## Abstract

**Background:**

The variability in the maxillary sinus anatomy makes dental implant planning challenging. One of the anatomical landmarks that could affect the decision for implant placement around the maxillary sinus is the sinus septa. This study aimed to retrospectively determine the prevalence, anatomical distribution, and morphology of the maxillary sinus septa.

**Materials and methods:**

This study included 309 CBCT images that were analyzed to determine the prevalence, height, location, and orientation of the maxillary sinus septa. Descriptive statistics, Mann‒Whitney U tests, and Kruskal‒Wallis tests were used for data analysis.

**Results:**

A total of 618 maxillary sinuses were analyzed. Maxillary septa were present in 30% (n = 188) of the sinuses and in approximately 45% of the analyzed images. The mean height of the septa was 5.09 mm. The presence of bilateral septa was evident in 49 subjects (35.25%). Female subjects were significantly more likely to have only one septum (n = 67, 53.6%, *p* < 0.05).

**Conclusion:**

The presence of septa is very common, found in one-third to approximately half of the evaluated cases, which warrants careful examination before any surgical interventions to avoid possible complications.

## Introduction

Dental implant surgery is used to restore functional dentition for both fully and partially edentulous patients. However, in cases with insufficient bone height in the posterior maxilla, the placement of dental implants becomes complicated by the presence of the maxillary sinus. Anatomically, the maxillary sinus comprises a pyramid-shaped cavity in the facial part of the skull, with its base lying on the lateral nasal wall and its apex extending up into the zygomatic process of the maxilla [1].

Pneumatization of the alveolus appears as the maxillary sinus develops. This pneumatization enhances the proximity of the alveolar crest to the maxillary sinus and varies greatly from side to side [2]. In addition, alveolar ridge resorption as a consequence of tooth extraction, pathology, or trauma also enhances this proximity. The maxillary sinus further expands inferiorly in edentulous patients, usually filling a large part of the alveolar process. In extreme cases, long-term edentulousness can leave a paper-thin bone wall separating the maxillary sinus from the oral cavity [3]. Dental implant surgery in an atrophic posterior maxilla becomes more challenging as the maxillary bone height is reduced.

Sinus floor elevation and grafting are some of the most frequently used surgical interventions for obtaining adequate bone height before dental implant placement. The sinus lift technique was first presented by Tatum in the 1970s for implant placement in the atrophic posterior maxilla. Boyne and James 1980 [2] reported the lateral approach where a bone graft material is placed after forming a hinged door on the lateral sinus wall and reflecting the maxillary sinus membrane (Schneiderian membrane). However, this type of surgery has been reported to be prone to several complications, such as oro-antral fistulas, sinusitis, and sinus mucosal perforation [5–13]. Perforation of the maxillary sinus membrane was found to be the most common complication, with a prevalence of 44% [14–16].

The presence of anatomical variations, such as sinus septa, is considered one of the causes of this high perforation rate of the maxillary sinus membrane [16]. Irinakis et al. found the incidence of membrane perforation to be 22.8% and found a significant correlation between the presence of an interfering septum on cone beam computed tomography (CBCT) scans and the occurrence of sinus membrane perforation [17].

Maxillary sinus septa are bony crests inside the sinus. They can present in other paranasal sinuses as in frontal and sphenoid sinuses [18, 19]. They were first described by Underwood [20] in 1910 as walls of cortical bone inside the maxillary sinus that have an inverted gothic arch shape starting from the lateral or inferior wall of the sinus and they could even divide the sinus into two or more compartments.

The etiological development of maxillary sinus septa has been hypothesized by several authors. Underwood [20] proposed that septa are formed as a result of different eruption phases of the teeth. Neivert [21] suggested that septa are derived from the finger-like projections produced by the embryologic out-pouching of the ethmoid infundibulum, which the adjacent walls failed to absorb. However, Literature did not find a direct effect between the presence of septa and sinus functions such as immune defense by the mucociliary clearance, or production of nitric oxide[22].

Moreover, Krenmair et al. [23] classified septa into primary and secondary septa. The primary septa arise during the development of the maxilla, while the secondary septa arise from the irregular pneumatization of the maxillary sinus floor following tooth loss. The maxillary sinus septum was further classified according to the direction of the septum on CBCT. Class I: The septum is positioned in a buccal-lingual direction. Class II: the septum is positioned in a mesial-distal direction. Class III: the septum is positioned horizontally (shelf-like), and Class IV: the septum shows a combination of Classes I, II, or III. [17].

The maxillary sinus septa vary in number, length, and thickness, which could influence the surgical placement of dental implants in the sinus augmented posterior maxilla. The presence of a septum could limit the creation of a window in the lateral maxillary sinus wall and consequently the reflection of the hinged door. Lateral window surgery is a complicated technique, and in the presence of maxillary sinus septa, the maxillary sinus membrane is more susceptible to perforation during elevation [3, 12, 24–26]. Thus, evaluating this anatomical variation is essential when planning sinus graft surgery to prevent sinus membrane perforation.

Evaluation of the anatomical structures in the maxillary sinus is essential for the success of sinus lift surgical procedures. Therefore, a precise and exact definitive assessment is required [27, 28]. Many radiographic modalities have been used to identify maxillary sinus septa, such as dental panoramic radiography, computed tomography (CT) and cone beam computed tomography (CBCT) [6, 23, 24, 27–34]. During the last decade, CBCT has been proposed for maxillofacial imaging. It was first reported in the literature by Mozzo et al. [35] and has been recommended for evaluating anatomical structures, as it is considered an excellent modality with a low cost and reduced radiation exposure compared to classic CT scans [4, 36–38].

According to the literature, the prevalence of sinus septa varies from 16 to 58% [20, 23, 24, 39, 40]. The first study on the sinus septa performed in the Middle East was conducted in Iran, and it showed the prevalence of sinus septa to be 58% in the Iranian population [40].

Several studies investigated the population of Saudi Arabia, particularly in the Qassim region and at King Abdulaziz University in Jeddah, and found prevalences of sinus septa of 37.64% and 46%, respectively [41, 42]. In the Riyadh region, a study conducted of different incidental findings in maxillary sinuses found that 5% of the examined cone-beam CT scans had septa [43].

The purpose of this study was to retrospectively determine the prevalence, anatomical distribution, and morphology of maxillary sinus septa and to compare edentulous patients with dentate patients who attended Kind Saud University Dental College in Saudi Arabia.

## Materials and methods

This retrospective cross-sectional study was approved by the Institutional Review Board (IRB) of King Saud University Medical City (KSUMC) E-20–4695.

Cone-beam computed tomography (CBCT) scans were obtained from the Radiology Department at Dental University Hospital, King Saud University. The inclusion criteria for the CBCT scans were: patients aged 18 years or above, Saudi nationality, absence of artifacts, scans showing the bilateral maxillary sinuses, and both sides of the entire maxillary sinuses clearly visible. Patients with pathological changes in the maxillary sinus or segmented maxillary sinus images were excluded from the study.

Scans were evaluated to assess the prevalence of septa, considering patient age and sex as well as the number and orientation of septa and their locations. If septa were present, their height was measured and their orientation was determined. The septa were considered for analysis if they measured at least 1 mm.

**Fig. 1:**
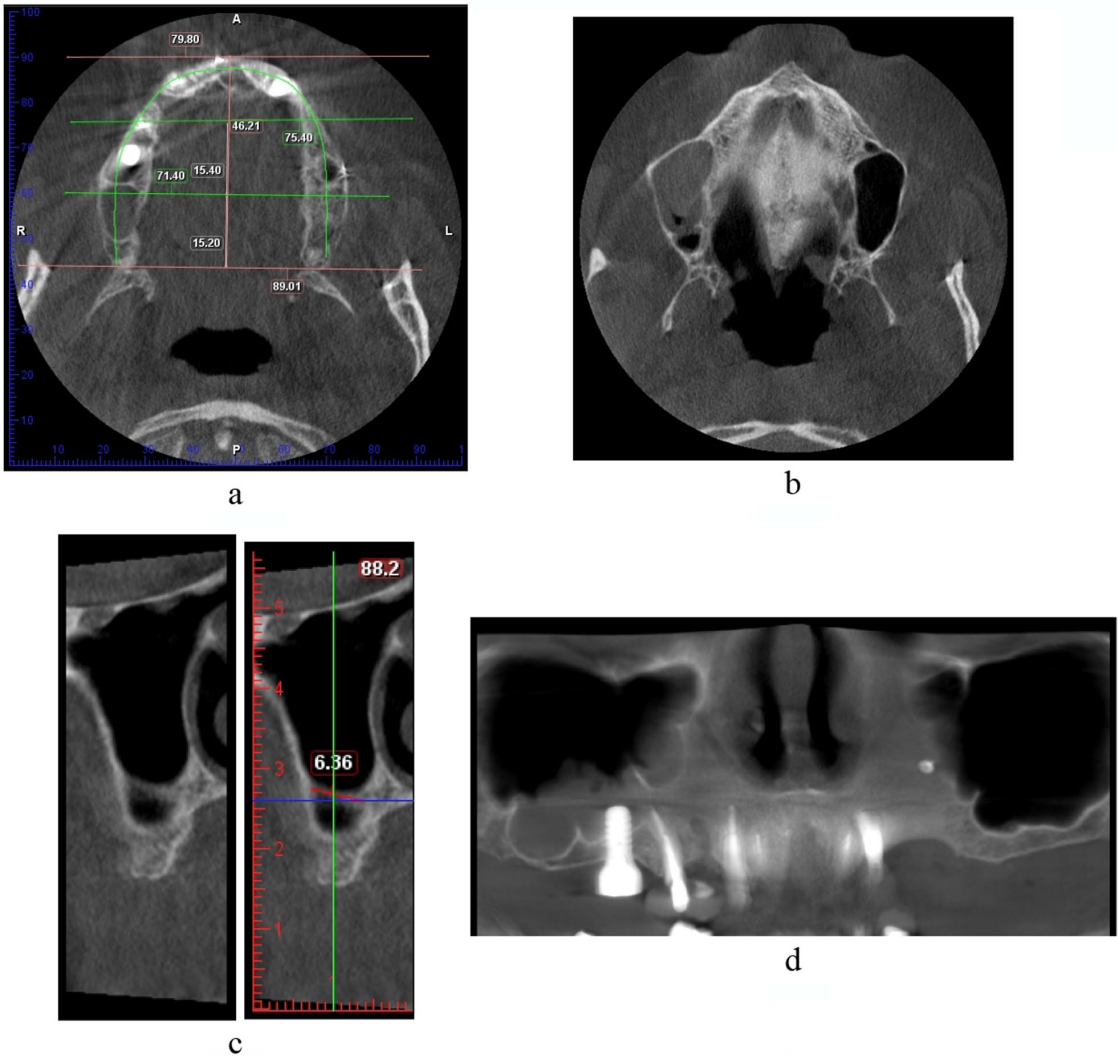
CBCT sections of a dentate patient. **a** Axial section where the maxillary jaw is divided into three sections. **b** Detection of a septum in the middle third of the sinus. **c** Cross-sectional cuts showing the orientation and length of the detected septum (with and without measurement). **d** Panoramic view showing multiple septa in the lower compartment. *Note* the detection of the other two septa in the medial wall of the sinus.

The evaluations of the maxillary sinus septum morphology, location, and prevalence were carried out in three sections:

1. Axial section (Fig. 1a, b): In this section, the maxilla was equally divided into anterior, middle, and posterior segments to determine the location of the septa in relation to the maxilla. This was done by drawing a horizontal line in the axial image connecting the most posterior part of the tuberosity. Then, a perpendicular line was drawn on the abovementioned line dividing it and connecting it to the most anterior part of the maxillary arch. The length of this perpendicular line was then divided by 3, which is the length of each section of the maxilla.
2. In the cross-sectional section (Fig. 1c), the orientation and length of the sinus septa were detected from cross-sectional images:
  The orientation types are 1. Horizontal: if the septum is emerging horizontally in the sinus from the lateral wall of the sinus, 2. Vertical: if it is oriented vertically from the lateral wall of the sinus, 3. Oblique: when the septum is neither vertically nor horizontally oriented. This was assessed on the axial sections as well.
3. The panoramic section (Fig. 1d): The septal height in the sinus was assessed in this section. Each sinus was divided into two compartments by a horizontal line placed in the middle of the sinus: the upper and lower compartments.

To obtain a precise measurement of the sinus septal height, the thickness of the reconstructed images was set as 1 mm for all three sections.

All measurements were accomplished with software programmed for an automated image measuring tool, Romexis software (Planmeca Romexis, Planmeca, USA).

The examinations and measurements were performed by two experienced and trained periodontists (AM, RA). For inter- and intraexaminer reliability, the measurements were performed 3 times by the same observers. The same observer also repeated the measurements twice at an interval of 2 weeks.

### Statistical analysis

Statistical analyses were carried out using SPSS version 22.0.1 (SPSS, Chicago, Ill., USA). To assess the intraobserver reliability, the Wilcoxon matched-pairs signed-ranks test was used for repeated measurements by the same observer. Independent groups in the study were compared using the Mann‒Whitney U test; the results are expressed as the means and standard deviation. Nonparametric data were compared using the Kruskal‒Wallis test. The Pearson Χ^2^ test was performed for statistical analyses among sex, age, localization, and measurements (*p* < 0.05).

## Results

A total of 314 CBCT scans were initially obtained, among which only 309 patients’ CBCT scans met the inclusion criteria, with a total of 618 maxillary sinuses. The interexaminer and intraexaminer reliabilities were 0.8 and 0.93, respectively.

The age of the included patients ranged between 18 and 84 years, with most of the patients (approximately 29%) being 21–30 years old. Female subjects represented 71.52% (n = 221) of the sample.

### Prevalence of septa

Septa were present in 139 subjects (44.98%), with 203 septa in 188 sinuses (30.4%). The distribution of septa is presented in Tables 1, 2 and 3.

**Table 1:** Septa distribution in the patient population

	Total no. of subjects (%)	Subjects with septa (%)*	Total number of septa per group %*
*Sex*			
Male	88 (28.47%)	45 (51.14%)	78 (38.24)
Female	221 (71.52%)	94 (42.5%)	125 (61.57)
*Location*			
Septa present in a unilateral sinus	90 (29.13%)	90 (64.2%)	94 (46.31)
Septa present in bilateral sinuses	49 (15.86%)	49 (35.25%)	109 (53.7)
*Age group*			
Less than 20	14 (4.53%)	3 (21.42%)	4 (1.97)
21–30 years	89 (28.8%)	39 (43.82%)	56 (27.6)
31–40 years	70 (22.65%)	33 (47.14%)	52(25.62)
41–50 years	52 (16.83%)	27 (51.9%)	40 (19.7)
51–60 years	39 (12.62%)	15 (38.46%)	19 (9.36)
More than 60 years	45 (14.65%)	22 (48.8%)	32 (15.76)
*Edentulism*			
Dentate	288 (93.2%)	128 (44.4%)	189 (93.1)
Complete edentulous	21 (6.8%)	11 (52.4%)	14 (6.9)

*Percentage is calculated according to the total septa count

**Table 2:** Distribution of septa per sinus M: number of male patients, F: number of female patients

Septa	Right sinus			Left sinus			Bilateral sinus			Total subjects		
	M	F	Total no. of patients	M	F	Total no. of patients	M	F	Total no. of patients	M	F	Total
One	9	16	25	10	51	61				19	67	86
two	1	1	2	2	0	2	16	24	40	19	25	44
Three							4	3	7	4	93	7
Four							2	–	2	2	–	2
Total septa	29			65			109					139
										203

**Table 3:** Distribution of septa by occurrence in maxillary sinuses per subject

Bilaterally symmetrical septa		Bilaterally non-symmetrical septa	
Single septum in each sinus	40	Single septum in one sinus only	86
2 septa in each sinus	1	2 septa in one sinus only	4
		Multiple septa in both sinuses	8
Total		139	

**Fig. 2:**
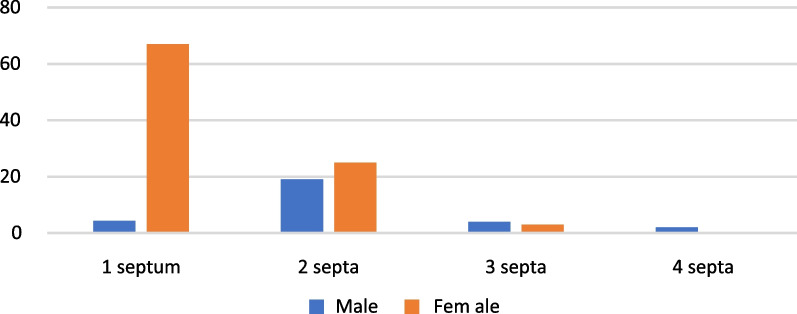
Frequency of septa per subject

The prevalence of septa in the included subjects according to sex and age was not statistically significant (*p* = 0.32 and *p* = 0.09, respectively). Female subjects were significantly more likely to have only one septum (n = 67, 53.6%, *p* < 0.05). In patients with septa, men tended to have multiple septa (*p* < 0.03). The presence of bilateral septa was evident in 49 subjects (35.25%). Approximately 65% of the subjects had unilateral septa rather than bilateral septa (*p* < 0.02). In subjects with bilateral septa, the unique presentation of one septum per sinus was significantly more common than any other presentations of bilaterally occurring septa (*p* < 0.002). The maximum number of septa in one subject was four, which were distributed bilaterally over both sinuses, and this was observed in only 2 male subjects (Fig. 2).

### Anatomical location of septa

**Fig. 3:**
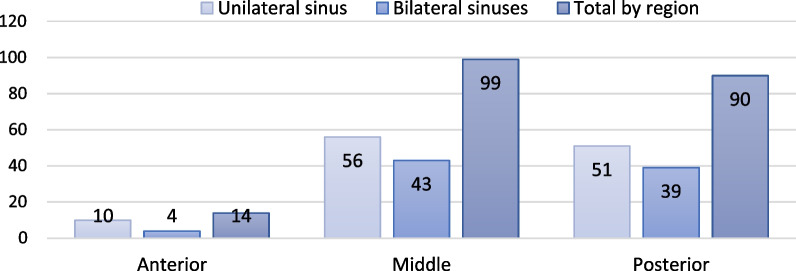
Septa distribution according to the anatomical location within the maxillary sinus

**Fig. 4:**
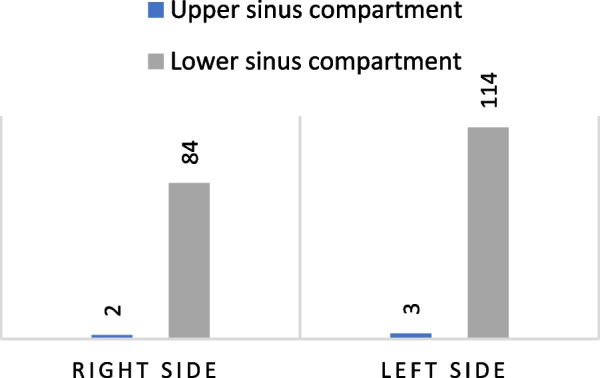
Septa distribution according to the upper and lower sinus compartments

A significant difference was found concerning the anatomical distribution of the septa in the sinus for septa located in the anterior region of the maxillary sinus compared to those located in the middle and posterior regions (p<0.006, p<0.007, respectively) (Fig. 3). Most septa were located in the middle part of the sinus, followed by the posterior sinus segment, with a minor statistically insignificant difference between the two (*p* = 0.45). More septa were located within the lower sinus compartment than within the upper compartment, and this was significant for both the left (*p* = 0.001) and right sides (*p* = 0.002) (Fig. 4).

### Size and morphology of the septa

The length of the septa ranged from 1.0 mm up to 17.99 mm, with an average length of 5.09 ± 2.4 mm. The length of approximately two-thirds of the detected septa was approximately 5 mm or less (Table 4).

**Table 4:** Septa length frequency and percentage

Septa length	Frequency	%
From 1 to 5	129	63.55
From 6 to 10	63	31.034
From 11 to 15	10	4.93
From 16 to 20	1	0.5
Total	203	100

A greater number of septa presented with complete extension in both the right and left sinus sides (*p* = 0.044, *p* = 0.007, respectively). For the partially extended septa, a higher number was associated with the vertical type of orientation on both sides (right: *p* = 0.044, left: *p* = 0.007, respectively) (Table 5).

**Table 5:** The morphology of the septa extension and orientation in relation to the sinus sides

	Extent		*P* value	Orientation (partial septa)		
	Complete	Partial		Horizontal	Vertical	Oblique
Right side	69	17	0.044	1	15	1
Left side	77	40	0.007	9	24	7
Total septa	146 (71.92%)	57 (28.1%)	0.014	10 (17.5%)	39 (68.4%)	8 (14.03%)

### Edentulous subjects

Out of the 309 subjects, twenty-one were completely edentulous (6.7%). Approximately half of the edentulous subjects presented with septa (5.4% of the total subjects), with a total of 14 septa (Table 6).

**Table 6:** Descriptive data of the edentulous subjects with septa

	Number of subjects	Total septa
*Sex*		
Male	4	5
female	7	9
*Age group*		
41–50 years	4	5
51–60 years	2	3
More than 60 years	5	6
*Location*		
Unilateral sinus	9	10
Bilateral sinuses	2	4
Total	11	14

**Fig. 5:**
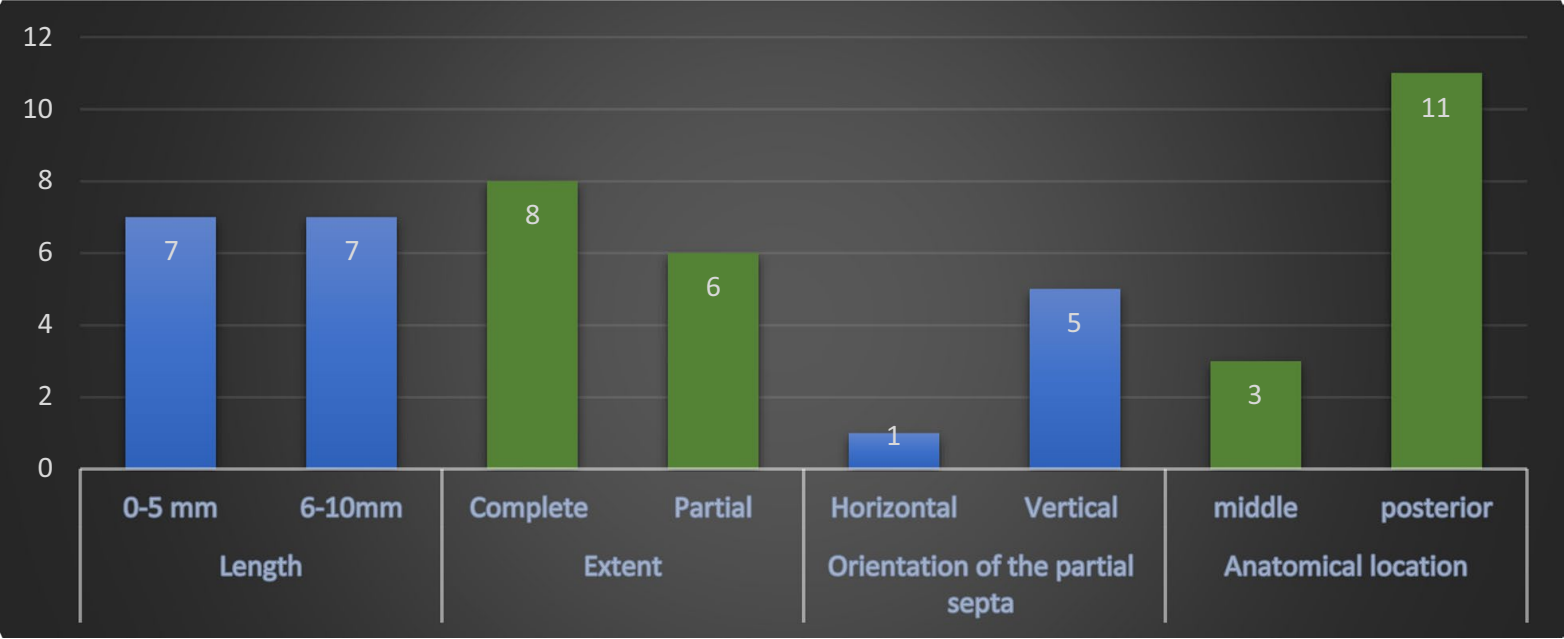
Septa characteristics in edentulous subjects

The anatomy and morphology of the septa in these subjects are presented in Fig. 5. All septa were present in the lower sinus compartment, with no significant difference regarding length and extent in comparison to the dentate patients. However, most of these septa were located posteriorly (*p* < 0.02).

## Discussion

This study aimed to evaluate the prevalence of maxillary sinus septum among patients attending dental clinics at King Saud University. The prevalence of maxillary sinus septa varied among previous studies. While some authors reported the prevalence to be as low as 16% [23], others reported it to be as high as 58.3% [44].

Other investigators presented a more specific estimate for the percentage of septa among patients and sinuses; for example, Bornstein and colleagues found septa to be present in 66.5% of patients and 56.5% of all evaluated sinuses [45]. Another study found septa in 44% of the patients and approximately 31.17% of the sinuses [46]. In a recent meta-analysis, the mean prevalence of septa per patient was 41%, while the prevalence by sinus was 33% [47]. The variability among the percentages reported can be attributed to the differences in patient samples, including their age and ethnicity; the use of various evaluation methods, such as cadaveric dissection, surgical evaluation, and radiographic examination; differences in the types of radiographs selected (2-D versus 3-D); as well as the variability of the septal inclusion criteria (i.e., the minimal height and thickness for their identification) [46, 47]. Our study found septa occurred in approximately 45% of the evaluated patients and approximately 30% of the evaluated sinuses, which is in line with the previous studies.

In this investigation, although more septa were found among female patients and the 21–30 age group had the highest number of septa, these findings failed to reach statistical significance, and the presence of septa was not considered to be associated with the patient's age or sex. While similar results were already reported by other authors [40, 47], our findings should be interpreted carefully, as our sample size might not allow us to sufficiently and reliably investigate these two factors.

Comparing the occurrence of septa in the right versus the left sinuses revealed no significant difference, which was in accordance with [48], where no statistically significant difference was detected when evaluating both sides (*p* > 0.05). In contrast, some differences were reported in other studies with no clear explanation for the reasons [40].

Our study presented a lower number of subjects with bilateral septa (only 35.25% of all patients with septa had them bilaterally). However, the total number of septa was higher in the bilateral septa group. Similarly, most septa were found to be unilateral in approximately 58.3% and 55% of patients in the study of Toprac and Atac and the study of Takeda et al., respectively[46, 49].

Regarding septal location, the current evaluation found septa to most commonly be present in the middle region of the sinus, followed by the posterior sinus segment, with a statistically nonsignificant difference. This was in agreement with several other studies; for example, Furtado and his colleagues found the highest percentage of septa to be in the middle sinus region (44.2%) [48], while Toprac and Atac found it was 59.62% [46], and the Orhan group reported 69.1% [40]. Meanwhile, contradictory results were also reported by Schriber and his group, who found the majority of septa to be located in the posterior maxilla in the region of the second molars [50], whereas Shahidi et al. found most septa were in the anterior region (Shahidi et al. [40]) and [51] reported no significant difference among the locations of septal occurrence in the anteroposterior plane [51]. Again, these variations can be explained by the differences in the studied populations, where age groups and sequence of dental extractions can vary, affecting the pneumatization [46].

The mean height of the septa in this study was approximately 5 mm, and other studies reported that the average length reached 6.34 ± 3.05 mm [46] and 7.5 mm [52]. Knowledge of the average septal height might affect decisions about the mode of management of septa during sinus lift surgeries.

Most septa detected in this study were of the complete type, i.e., dividing the sinus into separate compartments. Similar results were reported in the study of Furtado, with 59.6% of the septa being complete [48]. On the other hand, a lower prevalence of complete septa was reported by multiple other authors, such as Shahidi et al. (25.2%) [39] and Pommer et al. (0.3%) [52].

Edentulism is commonly associated with sinus expansion, and thus, its relationship with the presence of septa was investigated. The study by Toprac and Atac [46] found most of the septa to be present in completely edentulous patients (50.96%). This was explained by the emergence of what are defined as “secondary septa” after dental extractions. Despite the small number of edentulous patients included, our study failed to identify any relationship between dentate status and the presence of septa. Similarly, the study by Schriber et al. supported the concept of a lack of an effect of dentate status on septa prevalence [50]. Surprisingly, the study by Orhan reported the highest septa prevalence in partially edentulous subjects compared to both dentate and completely edentulous subjects [40]. One possible explanation for this variability is that the inclusion of young children in that study with mixed dentition caused this variation in the findings.

## Conclusion

The results of the current study indicate that the presence of septa is very common, found in one-third to approximately half of the evaluated cases, which warrants careful examination before any surgical interventions to avoid possible complications. It is still not clear which factors can affect their presence and whether the number or size of septa can change over time. Thus, additional studies evaluating them over time are recommended.

## Data Availability

The datasets analyzed during the current study are available from the corresponding author on reasonable request.
